# Analysis of the practice of switch of antibiotics from intravenous to oral therapy at a tertiary care hospital in Nepal: a prospective observational study

**DOI:** 10.1017/ash.2024.494

**Published:** 2025-01-27

**Authors:** Upasana Acharya, Sweta Shrestha, Aastha Rawal, Laxmi Dangol, Binaya Sapkota

**Affiliations:** 1 Department of Critical Care Medicine, Grande International Hospital, Tokha, Kathmandu, Nepal; 2 Department of Pharmacy, Kathmandu University, Dhulikhel, Kavre, Nepal; 3 Department of Infection Control, Manmohan Memorial Medical College and Teaching Hospital, Swoyambhu, Kathmandu, Nepal; 4 Department of Infection Prevention and Control, Grande International Hospital, Tokha, Kathmandu, Nepal; 5 Department of Pharmaceutical Sciences, Nobel College Faculty of Health Sciences, Sinamangal, Kathmandu, Nepal

## Abstract

**Objective:**

This study analyzed the practice of switching intravenous antibiotics to oral dosage form in a tertiary care hospital of Nepal.

**Design:**

A prospective observational study was performed among patients admitted to medical/surgical wards in a private tertiary care hospital of Nepal.

**Methods:**

Hospitalized adult patients who received IV antibiotics for at least 48 hours and met the eligibility criteria were enrolled in the study. The detailed information on use of antibiotics such as indication, duration, type time of switch etc. were collected and analyzed.

**Results:**

Among 335 patients, 282(83.9%) met the eligibility criteria for intravenous (IV) to oral conversion however, only 18.7% of patients were switched to oral regimen. Step-down conversion was the common type of IV to oral switch. Almost all patients were empirically treated with β-lactams antibiotics (n = 327). There was significant association between the antibiotic class and duration of IV therapy (*P* < 0.001). The length of hospital stays and duration of IV antibiotics therapy was lower in the timely converted group than in the non-converted groups (*P* < 0.001). The duration of IV antibiotics therapy was strongly correlated with duration of hospital stay (*r* = 0.743, *P* < 0.001).

**Conclusion:**

The findings revealed a low prevalence of conversion from IV antibiotics to oral, despite a higher percentage of patients meeting the eligibility criteria for conversion. There is a need for the implementation of structured program to review patients on IV antibiotics and promote timely conversion to oral once they meet the conversion criteria.

## Introduction

Antibiotics are one of the most widely but injudiciously used classes of medicines all over the world.^1^ The use of antibiotics is largely unregulated in developing countries.^2,3^ Many factors need to be considered while prescribing and dispensing antibiotics appropriately such as characteristics of antibiotics (e.g., bioavailability, pharmacokinetics, adverse drug effects), host factors (e.g., severity, organ function), switching of empiric to definitive treatment, and conversion of parenteral to oral for the eligible candidates in the shortest possible duration.^4^ The decision regarding the route of administration of antibiotics depend on several factors like patient’s condition and site of infection. Selecting the most appropriate route of administration is a part of the quality use of medicines.^5,6^

Parenteral antibiotic use is reportedly higher in many countries. Parenteral administration is most common in countries in West and Central Asia, Latin America, and Eastern and Southern Europe, where it accounted for more than 80% of patients on antibiotics.^7,8^ Inappropriate antibiotic prescription is also high in Nepal with a higher share of parenteral administration of the same.^9–12^ A substantial proportion of patients who fulfill the eligibility criteria supporting an early intravenous (IV) to oral switch are usually switched to oral therapy from their corresponding IV antibiotics at the time of discharge from the hospital.^13,14^ Intravenous therapy of shorter duration (2–3 days) pursued by oral treatment is advocated by several authors and practitioners acknowledging the potential advantages of this practice such as decreased risk of catheter-related infections and healthcare expenditures, increased patient convenience, and possibility of earlier hospital discharge.^15–19^ Additionally, availability of oral antibiotics offering good bioavailability and reliable absorption and efficacy have underpinned the notion of early IV to oral switch of therapy.^20,21^ However, IV to oral conversion of antimicrobials, which is an important facet of Antibiotic Stewardship Program, requires fulfillment of some important criteria such as patient should have functional gastrointestinal tract allowing adequate absorption of oral antibiotics, and should be clinically stable. Patients’ eligibility for the same is evaluated based on their white blood cell (WBC) count, temperature, and specified standard for specific infections.^15,20,21^ If these criteria permit, then they will be subjected to sequential conversion, switch conversion, or step-down conversion of antibiotics based on the clinical discretion of the physicians. Sequential conversion refers to replacing a parenteral version of a medication with its oral counterpart of the same compound. Switch conversion describes the conversion of an IV medication to an oral equivalent within the same class having same level of potency but of a different compound. Step-down conversion refers to the conversion from an injectable medication to an oral agent in another class or to a different medication within the same class where the frequency, dose, and the spectrum of activity may not be exactly the same.^21^

Despite advances in IV to oral conversion practice, the scenario is still not up to the standard in developing countries like Nepal where implementation of antimicrobial stewardship programs and frequent change in antimicrobial prescribing habits without rational antimicrobial susceptibility testing is a common phenomenon.^3,22^ Though there is a dire need to investigate the practice of antibiotic usage, IV to oral switch of antibiotics, no such study has yet been conducted in hospitals in Nepal. Hence, to void the knowledge gap in IV to oral antibiotics switch in the country, we conducted this study to assess the practice of IV to oral switching practice of antibiotics in a tertiary care hospital in Nepal. The situation might be replicable to similar healthcare settings within the country as well as in other developing countries.

## Methods

### Study design and study site

A prospective, observational study was conducted over a period of eight months from February to September 2021 in a tertiary care hospital named Grande International Hospital located at Tokha, Kathmandu. A hospital with 200-bed capacity has medical and surgical facilities, with an average of 64% of total bed occupancy and 500 patients daily visit on outpatient basis to get medical services from across the country.

### Sample size calculation

The sample size for this study was calculated using Cochran’s formula:

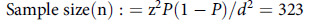




where n is the sample size, *Z* is the statistic corresponding to level of confidence,

*P* is expected prevalence (Prevalence of conversion from IV to oral therapy from pilot study was calculated to be 30%, and d is precision (corresponding to effect size).

Slightly more participants were taken to overcome drop out of patients and missed data. Hence, the final sample size taken for the study was 335.

## Study participants

The study population consisted of patients admitted to medical and surgical wards of the hospital. Consecutive sampling was done to include all hospitalized adult patients who received IV antibiotics for at least 48 hours within study period. The patients with physiologic condition rendering them ineligible for oral dosage form (e.g., malabsorption syndrome, partial or total removal of stomach, short bowel syndrome) and patients with malignancies were excluded from the study.

Eligibility criteria for IV to oral switch was defined as follows^21^:

- Patients who had received an IV antibiotic for ≥48 hours and were able to tolerate oral therapy
- No vomiting or diarrhea or nil per oral
- Clinical improvement with temperature <38°C
- Systolic blood pressure >90 mmHg
- Heart rate <100 beats per minute
- Normal WBC count or a decrease of at least 2000 cells/μL over the last 24 hour

### Data collection tool

A structured data collection form was developed after an extensive literature review and was discussed with a group of experts [Appendix 1]. The form consisted of four sections. The first section consisted of the sociodemographic characteristics of the patients, information on comorbidities, indication of antibiotic therapy, and duration of hospital stay. The second part included the details of the antibiotics administered (e.g., route of administration, duration of IV therapy, time of switch if applicable, duration of oral therapy, type of conversion applied). The third section covered the records of signs and symptoms and clinical parameters essential to assess the clinical stability of the patient. The fourth section included information on culture and sensitivity test, the specimens collected, and the microorganism isolated. This record was maintained daily until the date of discharge of the patient.

### Validation of data collection tool

For validity of self-designed data collection tool, pilot study was done on small group of similar study population (32 subjects, 10% of sample size) which was not included in the final analysis. Some minor changes were done on data collection tool after pilot study.

### Data collection

After obtaining approval from institutional review committee of Grande International Hospital (Reg.no. 01/ 2020), the clinical pharmacist in the hospital visited the wards daily and collected the information in the data collection sheet. All patients hospitalized for more than 24 hours from February to September 2021 were screened for inclusion, but only the patients who received IV antibiotics for more than 48 hours were included. The primary treating physician was not informed about the study undertaken to avoid the possibility of Hawthorne effect. The patients’ medical file and medication chart were followed until the time of discharge to obtain required information in such a way that patients were not directly involved in data collection. Any unclear information was confirmed with the assigned nurse in the respective ward.

### Statistical analysis

Statistical analysis of data was performed using Statistical Package for the Social Sciences version 26.0. Descriptive analysis was done to represent characteristics of patients using frequencies and percentages for categorical variables and mean and standard deviation for continuous variables. Inferential statistics like chi-square or Fisher test were used to show association between different categorical variables like type of conversion, duration of therapy, body system involved, different classes of antibiotics, etc. A *P*-value less than 0.05 was considered statistically significant.

## Results

A total of 2073 patient’s admission records were screened for inclusion, out of which 335 were enrolled for final analysis based on the eligibility criteria for the study. The characteristic of study sample is shown in Table 1. Out of 335 patients, 20 patients (5.97%) were on concurrent IV and per oral antibiotics and 315 (94.03%) were on IV antibiotics only.

**Table 1.** Characteristics of study sample (n = 335)

Variables	n (%)
**Age (in years) Mean ± SD**	**49.8 ± 18.7**
19–29	55 (16.4)
30–41	65 (19.4)
42–53	84 (25.1)
54–65	54 (16.1)
>65	77 (23)
**Gender**	
Male	174 (51.9)
Female	161 (48.1)
**Duration of hospital stay (days) (Mean ± SD)**	4.37 ± 1.98
**Comorbidity**	
Hypertension	61 (18.2)
Diabetes Mellitus	45 (13.4)
Hypothyroidism	17 (5.1)
Chronic kidney disease	13 (3.8)
Anemia	12 (3.6)
Chronic obstructive lung disease	11 (3.3)
**Admitted department**	
Orthopedic surgery	102 (30.4)
Gynecology and obstetrics surgery	49 (14.6)
Pulmonary medicine	41 (12.2)
Urinary surgery	37 (11)
Renal medicine	30 (9)
Gastrointestinal surgery	26 (7.8)
Gastrointestinal medicine	12 (3.6)
ENT surgery	11 (3.3)
General internal medicine	5 (1.5)
Cardiology medicine	4 (1.2)
Autoimmune disorder	3 (0.9)
Others	15 (4.5)

### Empiric use of antibiotics and IV to oral conversion

Almost all patients were empirically treated with β-lactams antibiotics (327, 97.6%). Patients received empirical treatment either as monotherapy (261, 77.9%), or combination of more than one IV antibiotics (76, 22.7%), cephalosporins (193, 39.3%) being the most prescribed antibiotics in the combination regimens. Out of 335 patients, 282(83.9%) met the eligibility criteria for IV to oral conversion however, only less than one-fourth (62, 18.7%) of the patients were switched to oral regimen at 48 hrs. Beta-lactam antibiotics were mostly converted to oral dosage forms, accounting for 77% of conversion followed by beta-lactam with macrolide or fluoroquinolone in 7.5% of conversion cases. The IV to oral conversion was statistically significant for β-lactams (*P*-value <0.001).

Of total, 53 patients (16.1%) were identified as not fulfilling the eligibility criteria for IV to oral conversion. The most common reason for not meeting the eligibility criteria was tachycardia (63, 98%) followed by WBC count persisting above the limit (10, 15.6%). Out of 335 cases, 267(79%) patients were switched to oral antibiotics from IV route by the time of discharge from the hospital. The majority (188, 56.1%) of the patients were switched to oral antibiotics within day 7 and only 17 (5%) patients remained on IV antibiotics for more than 7 days.

### Type of IV to oral conversion

Among three different types of switch for IV to oral (i.e., step-down, switch, and sequential), step-down was observed to be the most common type of IV to oral switch among patients who were converted to oral regimen at 48 hrs. of IV therapy and who met eligibility criteria for IV to oral regimen (Table 2).

**Table 2.** Type of IV to oral conversion among patients meeting inclusion criteria (at 48 hrs. of receiving IV therapy)

Type of conversion	Frequency (%)
Step down	55 (79.7)
Switch	7 (10.1)
Sequential	3 (4.4)
Discontinuation	4 (5.8)
Days of IV therapy (Mean ± SD)	1.95 ± 1.9
Days of hospital stay (Mean ± SD)	3.2 ± 1.96

When all patients initially on IV antibiotic therapy, were followed till the time of discharge, group of patients on monotherapy with IV beta lactams antibiotics was found to be more frequently converted to oral via step-down type of switch as shown in Table 3. Table 4 shows the choice of antibiotics for empiric therapy and post-empiric therapy. Ceftriaxone (n = 122) was the most common choice for empiric use whereas cefixime was the most common choice for switch from IV to oral followed by ciprofloxacin.

**Table 3.** Type of switch among different classes of antibiotics

Type of switch	Different class of Antibiotics (%)			
	Beta lactam	Beta lactam with one another group	Beta lactam with 2 other group	Fluoroquinolone
Sequential	0	1.8	0	0
Step down	52.8	22.6	3.7	1.8
Switch	9.4	0	1.8	0
Stop	1.8	0	0	0

**Table 4.** List of antibiotics prescribed for empiric therapy and after empiric therapy

Name of Antibiotic	Empirical therapy n (%)	Antibiotics switched after empiric therapy n (%)
Stop	–	64 (18.7)
Ceftriaxone	122 (36)	–
Piperacillin – Tazobactam	79 (23.1)	8 (2.3)
Cephazolin	78 (24)	4 (1.2)
Amoxicillin – Clavulanic acid	26 (8.2)	45 (13.2)
Metronidazole	24 (7)	–
Azithromycin	20 (5.8)	–
Meropenem	18 (5.3)	2 (0.6)
Amikacin/Gentamicin	17 (5)	1 (0.3)
Levofloxacin/Ciprofloxacin	14 (4.1)	1 (0.3)
Linezolid	3 (0.9)	1 (0.3)
Ampicillin – Sulbactam	3 (0.9)	–
Colistin	2 (0.6)	–
Cefoperazone – Sulbactam	2 (0.6)	–
Doxycycline	2 (0.6)	3 (0.9)
Clindamycin	1 (0.3)	–
Cefuroxime	1 (0.3)	2 (0.6)
Ceftriaxone – Tazobactam	1 (0.3)	11 (3.2)
Tigecycline	1 (0.3)	–
Cefixime	–	102 (29.8)
Cefadroxil	–	4 (1.2)
Ciprofloxacin	–	60 (17.5)
Cephalexin	–	36 (10.5)
Cefpodoxime	–	3 (0.9)
Nitrofurantoin	–	1 (0.3)
Flucloxacillin	–	2 (0.6)
Faropenem	–	4 (1.2)
Cotrimoxazole	–	1 (0.3)

Further,335 patients were divided into two groups based on timely conversion to oral therapy (i.e., converted group versus non-converted group) and were analyzed to know whether there was an association between the conversion and body system affected for which IV therapy was initiated. The chi-square analysis of the association showed that there was significant association among the IV to oral conversion and the body system affected as shown in Table 5. Moreover, it was revealed that there was significant association among the antibiotic class and duration of IV therapy (*P*-value <0.001). The association shows that the antibiotics class such third-generation cephalosporin, fluoroquinolones, penicillin with beta-lactamase inhibitors, and first-generation cephalosporin when initiated were likely to be continued for 2–6 days (Table 6).

**Table 5.** Association between IV to oral conversion with body system affected (n = 335)

Body system affected	Status of IV to oral conversion		*P*-value
	n (%) (converted)	n (%) (not converted)	
Gynecology surgery	20 (40.8)	29 (59.2)	<0.001
Urinary surgery	16 (43.2)	21 (56.8)	
Pulmonary medicine	7 (17.1)	34 (82.9)	
Orthopedic surgery	5 (4.9)	97 (95.1)	
Gastrointestinal surgery	5 (19.2)	21 (80.8)	
Nephrology medicine	3 (10)	27 (90)	
Gastrointestinal medicine	1 (8.3)	11 (91.7)	
Multisystem/internal medicine	1 (20)	4 (80)	
ENT surgery	2 (18.1)	9 (81.9)	
Skin surgery	1 (50)	1 (50)	
Others	8 (40)	12 (60)	

**Table 6.** Association among the empiric antibiotic class and duration of IV therapy (n = 335)

Type of switch	Duration of IV route (in days) (n,%)			Total (n,%)	χ2(df) value; *P*-value [Cramer V]
	2 – 6	7 – 11	12+		
Stop	52 (15.4)	17 (5)	0	69 (20.5)	86.663 (39); <0.001 [0.293]
3^rd^ generation cephalosporin	87 (25.8)	1 (0.3)	0	89 (26.4)	
3^rd^ generation cephalosporin and nitroimidazole	6 (1.8)	1 (0.3)	0	7 (2.1)	
3^rd^ generation cephalosporin and macrolide	1 (0.3)	0	0	1 (0.3)	
1^st^ generation cephalosporin	33 (9.8)	2 (0.6)	1 (0.3)	35 (10.7)	
Fluoroquinolone	43 (12.8)	2 (0.6)	0	50 (14.8)	
Cephalosporin with beta lactamase inhibitor	6 (1.8)	0	0	6 (1.8)	
Fluoroquinolone and nitroimidazole	3 (0.9)	0	0	3 (0.9)	
Penicillin with beta lactamase inhibitor	43 (12.8)	3 (0.9)	0	46 (13.9)	
Change to another IV antibiotic	1 (0.3)	2 (0.6)	0	3 (0.9)	
2^nd^ generation cephalosporin	8 (2.4)	2 (0.6)	0	9 (3)	
Carbapenem	4 (1.2)	1 (0.3)	0	5 (1.5)	
Oxazidione	1 (0.3)	3 (0.9)	0	4 (1.2)	
Other	5 (1.5)	1 (0.3)	0	7 (2.1)	
Total (n, %)	293 (86.9)	35 (10.4)	1 (0.3)	335 (100)	

### Comparison between converted versus non-converted group of patients on duration of stay and IV therapy

**Figure 1.**
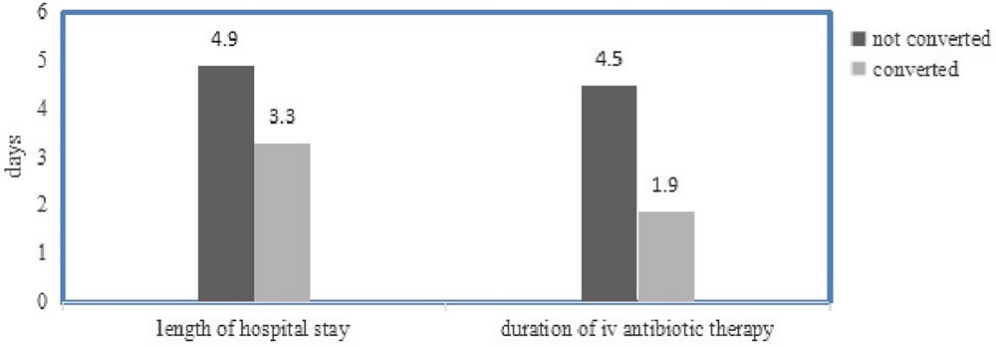
Length of hospital stay and duration of IV therapy in two groups.

There was statistical significance in both length of hospital stays and duration of IV therapy among the converted and the non-converted groups (*P* < 0.001). The correlation analysis showed that duration of IV therapy was strongly positively (*r*- value 0.743) correlated with duration of hospital stay and the correlation was significant (*P*-value <0.001) [Fig 1].

## Discussion

The status of IV to oral conversion of antibiotics is either neglected or undervalued in developing countries due to various reasons. Lack of human and institutional resources for the documentation, lack of stringent policy on antibiotics prescribing based on antimicrobial susceptibility pattern are few such reasons. The present study aimed to explore the status of IV to oral conversion of antibiotics in a tertiary care hospital in Nepal, which is often ignored dimension of study in clinical setting of developing countries.

Numerous international studies have demonstrated the advantages of switching antibiotics from IV to oral administration in hospital settings.^16–20^ These benefits array from the reduced hospital stays and cost-effectiveness to a decreased risk of infection due to IV lines and improved patient comfort. Despite these advantages, only 18.7% of the eligible patients underwent IV to oral conversion in our study, a rate lower than in studies conducted in Ethiopia^6^ (34.5%), Netherlands^13^ (54%), India^16^ (35%), and Switzerland^17^ (61.4%). However, this ratio aligns with another study conducted in Ethiopia.^23^ The physicians may hesitate to switch to oral antibiotics, despite meeting eligibility criteria for the same, because of lack of awareness about the benefits of early conversion or a lack of confidence in therapeutic efficacy of oral antibiotics compared to their IV counterparts.^23,24^ There are acceptable rates of timely conversion of IV to oral antibiotics in the Netherlands^13^ and Switzerland^17^ when interventions like implementation of guidelines for IV to oral conversion and the printed checklist of criteria for switching to oral antibiotics were introduced, respectively. Some studies have shown that clinical pharmacists played crucial role in timely conversion of IV antibiotics to oral medications after evaluating their eligibility criteria.^25–27^

We observed that the mean days for IV to oral conversion of antibiotics were 3.56, while the mean days for antibiotics use during hospital stay were 4.37±1.89, which were similar to the findings in India^16^ but significantly lower than the results from Ethiopia.^6^ Our study revealed beta-lactam antibiotics as the popular choice among the prescribers for both empirical and the converted therapies. Ceftriaxone was the most common choice for empiric therapy as in Ethiopian studies^6,23^ but ciprofloxacin was more commonly preferred during oral conversion. In our study, cefixime was the most common choice for oral conversion, sharing a similar spectrum with ceftriaxone. Another study by van Niekerk *et al.* (2012)^28^ showed amoxicillin-clavulanic acid as a common choice for switching to oral antibiotics. The preference for broad-spectrum beta-lactam antibiotics (ceftriaxone and cefixime) in both empiric and the converted therapy may indicate the presence of resistant microorganisms in our setting.

Both Tarekegn *et al.* (2022)^6^ and Shrayteh *et al*. (2014)^15^ revealed the sequential conversion as the most common type of conversion with fluoroquinolones. Conversely to the above finding, the most frequent type of conversion in our study is the switch followed by step-down approach. The variation in type of the conversion from IV to oral antibiotics may also be due to variation in following the operational definition of different switch types such as sequential, step-down, and switch.^21,29^ Ceftriaxone has no definitive oral equivalent, so conversion is mostly done through switch or step-down conversion therapy.

Regarding factors influencing IV to oral conversion, tachycardia was identified as a barrier, differing from the results in Ethiopia^6,23^ and Switzerland^17^ where tachypnea and fever/neutropenia were the most frequent barriers. Out of 335 cases in our study, 86% of switches were made within 2–6 days, comparable to the findings by Shrayteh *et al.* (2014).^15^ We found that 13(3.9%) received oral antibiotics in addition to IV antibiotics, a significantly lower rate than that reported by Berha and Kassie (2019) (45.07%).^23^ Observations revealed that in the converted group, the length of hospital stay is closely equal to duration of IV antibiotic therapy, supporting the idea that practicing physicians tend to halt IV therapy on the day of discharge. Numerous studies support that early switch from IV to oral antibiotics can reduce hospital stays and lower costs compared to conventional IV therapy.^17–19,30,31^

Timely conversion of antibiotics from IV to oral is a crucial component of antimicrobial stewardship programs, and the settings with such programs are expected to follow this practice in a timely manner. The observed low percentage may be attributed to a lack of in-house guidelines on such conversion and the clinicians’ lack of awareness of its benefits. Our results showed that more emphasis on IV to oral conversion should be placed on orthopedics and gastrointestinal medicine departments, which had a lower prevalence of conversion despite meeting eligibility criteria of the patients admitted.

While the benefits of the parenteral to oral conversion are evident, several challenges and considerations need to be addressed. The choice of antibiotic plays a crucial role in the successful conversion, which necessitates the choice of antibiotics with good oral bioavailability and a similar spectrum of activity. However, limitations may arise with certain antibiotics, such as those with poor oral absorption or limited oral equivalents. Furthermore, careful patient selection, consideration of pharmacokinetics, and interdisciplinary collaboration are crucial for the success of this conversion.

This study evaluated the practice of parenteral to oral conversion of antibiotics among the admitted patients in medical ward. Considering the fact that many antibiotics can be converted from IV to oral therapy, hospital must follow a structured program for reviewing the patients on IV antibiotics and encouraging timely conversion to oral once they meet the conversion criteria. Future research should focus on refining patient selection criteria, optimizing the conversion protocols, and evaluating the long-term outcomes to further enhance the implementation of parenteral to oral conversion programs in clinical practice. Clinical pharmacists can play a vital role in identifying and correcting the factors that prevent timely switch of IV antibiotics to oral therapy.

## References

[1] Klein EY , Van Boeckel TP , Martinez EM , et al. Global increase and geographic convergence in antibiotic consumption between 2000 and 2015. Proc Natl Acad Sci U S A 2018;115:E3463–E3470. 10.1073/pnas.1717295115 29581252 PMC5899442

[2] Holloway K , Dijk L van. The world medicines situation: rational use of medicines. World Med Situat 2011;2:24.

[3] Ghimire K , Banjara MR , Marasini BP , et al. Antibiotics prescription, dispensing practices and antibiotic resistance pattern in common pathogens in Nepal: a narrative review. Microbiol Insights 2023;16. 10.1177/11786361231167239 PMC10102948 37066121

[4] Leekha S , Terrell CL , Edson RS. General principles of antimicrobial therapy. Mayo Clin Proc 2011;86:156–167. 10.4065/mcp.2010.0639 21282489 PMC3031442

[5] McCarthy K , Avent M. Oral or intravenous antibiotics? Aust Prescr 2020;43:45–48. 10.18773/austprescr.2020.008 32346210 PMC7186270

[6] Tarekegn GY , Dagnew SB , Wondm SA , et al. Assessment of Intravenous Antibiotics to Peroral Antibiotics Conversion Practice and Its Associated Factor at University of Gondar Comprehensive Specialized Hospital: Prospective Observational Study. Can J Infect Dis Med Microbiol 2022;22. 10.1155/2022/8395424 PMC9581647 36277733

[7] Versporten A , Zarb P , Caniaux I , et al. Global-PPS network antimicrobial consumption and resistance in adult hospital inpatients in 53 countries: results of an internet-based global point prevalence survey. Lancet Glob Health 2018;6:e619–e629. 10.1016/S2214-109X(18)30186-4 29681513

[8] Labi AK , Obeng-Nkrumah N , Sunkwa-Mills G , et al. Antibiotic prescribing in paediatric inpatients in Ghana: a multi-centre point prevalence survey. BMC Pediatr 2018;18:391. 10.1186/s12887-018-1367-5 30572851 PMC6302438

[9] Baral P , Hann K , Pokhrel B , et al. Annual consumption of parenteral antibiotics in a tertiary hospital of Nepal, 2017-2019: a cross-sectional study. Public Health Action 2021;11(Suppl 1):52–57. 10.5588/pha.21.0043 34778016 PMC8575388

[10] Dixit SM , Shrestha B. Antibiotic prescribing pattern in different clinical departments at Kathmandu Medical College Teaching Hospital. J Kathmandu Med Coll 2018;23. 10.3126/jkmc.v7i1.20624

[11] Shankar PR , Partha P , Shenoy NK , et al. Prescribing patterns of antibiotics and sensitivity patterns of common microorganisms in the Internal Medicine ward of a teaching hospital in Western Nepal: a prospective study. Ann Clin Microbiol Antimicrob 2003;2:7. 10.1186/1476-0711-2-7 12904265 PMC179888

[12] Gyawali S , Shankar PR , Saha A , Mohan L. Study of prescription of injectable drugs and intravenous fluids to inpatients in a teaching hospital in Western Nepal. Mcgill J Med 2009;12:13–20. 19753281 PMC2687907

[13] Sevinç F , Prins JM , Koopmans RP , et al. Early switch from intravenous to oral antibiotics: guidelines and implementation in a large teaching hospital. J Antimicrob Chemother 1999;43:601–606. 10.1093/jac/43.4.601 10350396

[14] McLaughlin CM , Bodasing N , Boyter AC , et al. Pharmacy-implemented guidelines on switching from intravenous to oral antibiotics: an intervention study. QJM 2005;98:745–752. 10.1093/qjmed/hci114 16126741

[15] Shrayteh ZM , Rahal MK , Malaeb DN. Practice of switch from intravenous to oral antibiotics. Springerplus 2014;3:717. 10.1186/2193-1801-3-717 25674457 PMC4320166

[16] Tejaswini YS , Challa SR , Nalla KS , et al. Practice of intravenous to oral conversion of antibiotics and its influence on length of stay at a tertiary care hospital: a prospective study. J Clin of Diagn Res 2018;12:FC01–FC04. 10.7860/JCDR/2018/31647/11246

[17] Mertz D , Koller M , Haller P , et al. Outcomes of early switching from intravenous to oral antibiotics on medical wards. J Antimicrob Chemother 2009;64:188–199. 10.1093/jac/dkp131 19401304 PMC2692500

[18] Chandrasekhar D , Pokka Vayalil, V. Cost minimization analysis on IV to oral conversion of antimicrobial agent by the clinical pharmacist intervention. Clin Epidemiol Global Health 2019;7:60–65. 10.1016/j.cegh.2018.01.001

[19] Ehrenkranz NJ , Nerenberg DE , Shultz JM , Slater KC. Intervention to discontinue parenteral antimicrobial therapy in patients hospitalized with pulmonary infections: effect on shortening patient stay [published correction appears in Infect Control Hosp Epidemiol 1992 Mar;13(3):136]. Infect Control Hosp Epidemiol 1992;13:21–32. 10.1086/646419 1580920

[20] Mouwen AMA , Dijkstra JA , Jong E , et al. Early switching of antibiotic therapy from intravenous to oral using a combination of education, pocket-sized cards and switch advice: a practical intervention resulting in reduced length of hospital stay. Int J Antimicrob Agents 2020;55:105769. 10.1016/j.ijantimicag.2019.07.020 31362046

[21] Cyriac JM , James E. Switch over from intravenous to oral therapy: a concise overview. J Pharmacol Pharmacother 2014;5:83–87. 10.4103/0976-500X.130042 24799810 PMC4008927

[22] Otaigbe II , Elikwu JC. Drivers of inappropriate antibiotic use in low- and middle-income countries. JAC-Antimicrob Resist 2023;5. 10.1093/jacamr/dlad062 PMC10230568 37265987

[23] Beyene Berha A , Kassie GM. Current practice and barriers to an early antimicrobial conversion from intravenous to oral among hospitalized patients at Jimma university specialized hospital: prospective observational study. Interdiscip Perspect Infect Dis 2019;2019:7847354. 10.1155/2019/7847354 30867664 PMC6379851

[24] Engel MF , Postma DF , Hulscher MEJL , et al. Barriers to an early switch from intravenous to oral antibiotic therapy in hospitalised patients with CAP. Eur Respir J 2013;41:123–130. 10.1183/09031936.00029412 22653769

[25] Polidori P , Leonardi Vinci D , Adami S , et al. Role of the hospital pharmacist in an Italian antimicrobial stewardship programme. Eur J Hosp Pharm 2022;29:95–100. 10.1136/ejhpharm-2020-002242 32900820 PMC8899682

[26] Chikeka K , Gbadamosi K , Tran H , et al. Pilot study: impact of a pharmacist-led 48-hour antibiotic time-out on antibiotic utilization and outcome measures in a community teaching hospital. Hosp Pharm 2023;58:277–281. 10.1177/00185787221139429. 37216074 PMC10192984

[27] Hunter KA , Dormaier GK. Pharmacist-managed intravenous to oral step-down program. Clin Ther 1995;17:534–516. 10.1016/0149-2918(95)80119-7 7585857

[28] van Niekerk AC , Venter DJ , Boschmans SA. Implementation of intravenous to oral antibiotic switch therapy guidelines in the general medical wards of a tertiary-level hospital in South Africa. J Antimicrob Chemother 2012;67:756–762. 10.1093/jac/dkr526 22167244

[29] Kuper K. Intravenous to oral therapy conversion. In: LB Murdaugh (ed), Text Book of Competence Assessment Tools for Health-System Pharmacies, 4th edition. ASHP;2008:347–360. 10.37573/9781585284030.031

[30] Tefera GM , Sileshi T , Mekete MD , et al. Opportunities, associations, and impact of early intravenous to oral antimicrobial switch for hospitalized patients in Ethiopia. SAGE Open Med 2023;11. 10.1177/20503121231161192 PMC10064463 37008686

[31] Oosterheert JJ , Bonten MJ , Schneider MM , et al. Effectiveness of early switch from intravenous to oral antibiotics in severe community acquired pneumonia: multicentre randomised trial. BMJ 2006;333:1193. 10.1136/bmj.38993.560984 17090560 PMC1693658

